# Expression of Multiple Resistance Genes Enhances Tolerance to Environmental Stressors in Transgenic Poplar (*Populus* × *euramericana* ‘Guariento’)

**DOI:** 10.1371/journal.pone.0024614

**Published:** 2011-09-09

**Authors:** Xiaohua Su, Yanguang Chu, Huan Li, Yingjie Hou, Bingyu Zhang, Qinjun Huang, Zanmin Hu, Rongfeng Huang, Yingchuan Tian

**Affiliations:** 1 Key Laboratory of Tree Breeding and Cultivation, State Forestry Administration, Research Institute of Forestry, Chinese Academy of Forestry, Beijing, China; 2 Institute of Genetics and Developmental Biology, Chinese Academy of Sciences, Beijing, China; 3 Biotechnology Research Institute, Chinese Academy of Agricultural Sciences, Beijing, China; 4 Institute of Microbiology, Chinese Academy of Sciences, Beijing, China; Auburn University, United States of America

## Abstract

Commercial and non-commercial plants face a variety of environmental stressors that often cannot be controlled. In this study, transgenic hybrid poplar (*Populus* × *euramericana* ‘Guariento’) harboring five effector genes (*vgb*, *SacB*, *JERF36*, *BtCry3A* and *OC-I*) were subjected to drought, salinity, waterlogging and insect stressors in greenhouse or laboratory conditions. Field trials were also conducted to investigate long-term effects of transgenic trees on insects and salt tolerance in the transformants. In greenhouse studies, two transgenic lines D5-20 and D5-21 showed improved growth, as evidenced by greater height and basal diameter increments and total biomass relative to the control plants after drought or salt stress treatments. The improved tolerance to drought and salt was primarily attributed to greater instantaneous water use efficiency (WUEi) in the transgenic trees. The chlorophyll concentrations tended to be higher in the transgenic lines under drought or saline conditions. Transformed trees in drought conditions accumulated more fructan and proline and had increased *Fv*/*Fm* ratios (maximum quantum yield of photosystem II) under waterlogging stress. Insect-feeding assays in the laboratory revealed a higher total mortality rate and lower exuviation index of leaf beetle [*Plagiodera versicolora* (Laicharting)] larvae fed with D5-21 leaves, suggesting enhanced insect resistance in the transgenic poplar. In field trials, the dominance of targeted insects on 2-year-old D5-21 transgenic trees was substantially lower than that of the controls, indicating enhanced resistance to Coleoptera. The average height and DBH (diameter at breast height) of 2.5-year-old transgenic trees growing in naturally saline soil were 3.80% and 4.12% greater than those of the control trees, but these increases were not significant. These results suggested that multiple stress-resistance properties in important crop tree species could be simultaneously improved, although additional research is needed to fully understand the relationships between the altered phenotypes and the function of each transgene in multigene transformants.

## Introduction

The importance of trees on a regional and global scale cannot be overstated. In addition to their pivotal role in terrestrial and some aquatic ecosystems (e.g., mangroves), trees provide abundant commercial products, ranging from building materials to food and medicine [1], [2]. While many woody species have evolved a certain level of tolerance and resistance to environmental variables, an increasing number of stressors, such as drought, soil salinity, flooding and insects, threaten many species and entire populations. These stressors have the potential for reducing tree productivity and severely impacting the yield and quality of forest-derived materials, especially those from commercial plantations. Significant advancements in the use of genetic transformation methods in plant breeding research, particularly over the last decade, have allowed for the possibility of producing stress-tolerant tree varieties by utilizing proven genetic engineering techniques.

Studies based on single-gene transformation have generated large numbers of transgenic plants with enhanced tolerance to various environmental stresses, such as drought, salinity, waterlogging and insects, and have greatly improved our understanding of how plants cope with these adverse stimuli [3]–[5]. For example, osmoprotectants such as proline, glycine betaine, sucrose and fructan, produced by the expression of distinct genes, could help to moderate the adverse effects of drought and salt stress [3]. Introduction of the levansucrase gene, *SacB*, and the trehalose-6-phosphate synthase genes, *OtsA* and *OtsB*, was shown to enhance the tolerance of drought, salt or low temperature in transgenic *Beta vulgaris* (beet) or rice [6], [7]. Because transcriptional regulation is a crucial stress-response mechanism in plants, the genes that encode transcription factors are also promising targets for genetic engineering. Previous studies reported that overexpression of transcription factors, such as *CBF1* [8], *OsDREB1A* [9], *SNAC1* [10], *NF-YB* [11] and *DST* [12] enhanced stress tolerance in transgenic plants. The enzyme *Vitreoscilla* hemoglobin (VHb) produced by the aerobe *Vitreoscilla* under oxygen-limited conditions [13], such as those that may occur when soil becomes waterlogged after heavy rainfall, was shown to elevate intracellular oxygen levels and enhances the activity of terminal oxidases [14]. Expression of the VHb coding gene, *vgb,* improved growth in *Nicotiana tabaccum* [15], *Datura innoxia* [16] and *Petunia hybrida* Vilm [17]. *Bacillus thuringiensis* (Bt), which was shown to express proteins toxic to many insects, has been widely used to control crop pests [18]. Protease inhibitors and other proteins used alone, or combination with the *Bt* genes, could confer insect pest-resistance to important agricultural plants [19].

Expression or manipulation of multiple genes (so called ‘gene stacking’) to improve agronomic traits has been an advantageous approach for the development of genetically modified (GM) plants [20], [21]. To date, most gene transformations have been limited to 1–3 effector genes involved in the same or interconnected pathways. For example, Ye *et al*. [22] introduced genes related to the β-carotene biosynthetic pathway into ‘golden rice’ to produce provitamin A. Co-transformation of antisense *4CL* and sense *CAId5H* for lignin traits has been successfully performed in *Populus tremuloides* [23]. However, few studies have been reported on the enhancement of multiple pathways associated with a polygenetic trait by introduction of multigenes in forest trees. Since differential agronomic traits such as salt tolerance and insect resistance are commonly controlled by distinct genes, engineering of multiple genes that control distinct metabolic pathways would represent a significant progress toward the goal of simultaneous enhancement of multiple characteristics in woody species.

*Populus* × *euramericana* are interspecific hybrids between *Populus nigra* and *Populus deltoides*. Because the hybrids have demonstrated both rapid growth and easy vegetative propagation, many *P.* × *euramericana* cultivars have achieved commercial popularity and have been utilized by the forestry industry to enhance ecosystem stability. Through the use of biolistic bombardment mediated co-transformation, we previously obtained a multigene-transformed *P.* × *euramericana* hybrid, ‘Guariento’, an elite genotype widely planted in China [24]. Some of the transgenic lines were confirmed to have five effector genes: *vgb*, encoding aerobe *Vitreoscilla* hemoglobin; *SacB*, encoding levansucrase involved in fructan biosynthesis in *Bacillus subtilis*; *BtCry3A*, encoding δ-endotoxin from *B. thuringiensis* toxic to Coleoptera; *OC-I*, an insect-resistance gene encoding the proteinase inhibitor oryzacystatin I from rice; and *JERF36*, a tomato gene encoding jasmonate/ethylene-responsive factor protein. In this study, we investigated the growth and physiological responses to drought, salt and waterlogging stress in two transgenic lines (D5-20 and D5-21) harboring all five genes in greenhouse studies. Insect resistance in the transgenic plants was assayed in the laboratory, and field trials were also conducted to evaluate the effects of these transgenes on the insect community and salt tolerance.

## Results

### Expression of foreign genes in transgenic poplar

**Figure 1:**
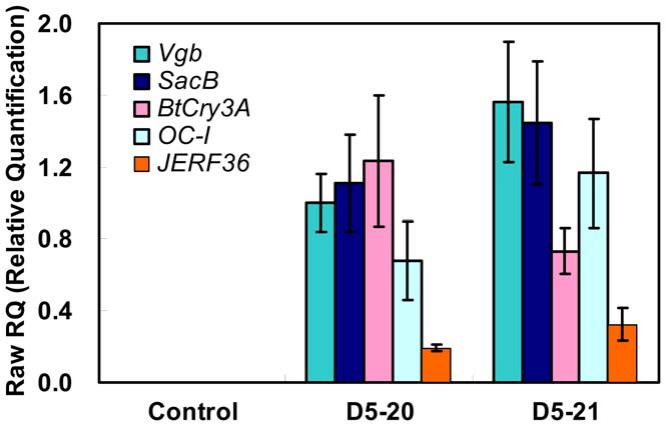
qRT-PCR analysis of relative expression levels of foreign genes of transgenic poplar. Poplar *ACTIN1* was detected as a reference gene.

The expression of each foreign gene was detected by quantitative real-time PCR (qRT-PCR) in the D5-20 and D5-21 transgenic lines, which displayed distinct profiles (Figure 1). The expression levels of *vgb* and *SacB* were high, while *JERF36* was relatively lowly expressed in both transgenic lines. Transcripts of *BtCry3A* and *OC-I* were detected at high levels in D5-20 and D5-21, respectively.

### Increased tolerance of transgenic poplar to drought stress

**Figure 2:**
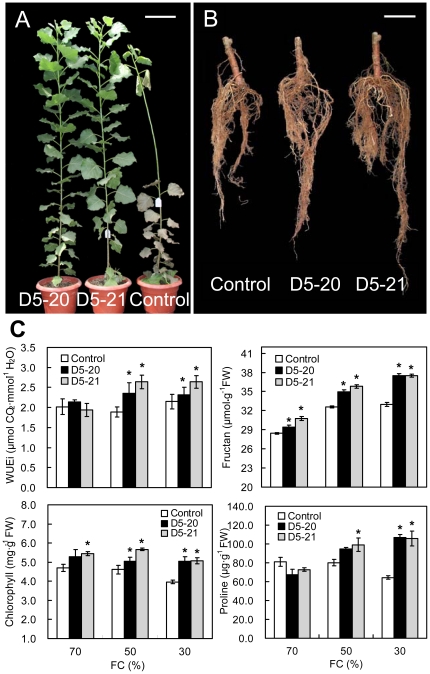
Improved drought tolerance of transgenic poplar overexpressing multiple resistance genes. Transgenic lines grew better with greener leaves (A) and had more extensive root systems (B) than the control after 76 days of drought stress (50% field capacity, FC). Only one representative tree for each line is shown. Bar: 20 cm (A), 10 cm (B). (C) Comparisons of instantaneous water use efficiency (WUEi) (upper left, n  =  9 plants), fructan levels (upper right, n  =  9 experiments), chlorophyll contents (lower left, n  =  9 experiments) and proline concentrations (lower right, n  =  9 experiments) of mature leaves from the transformants and the control exposed to different drought stresses for 76 days. FW  =  fresh weight. Data are means ± standard errors (SE). Asterisks indicate significant differences from the control (* *P* < 0.05).

At the end of the drought experiments, the multigene transformed poplar trees showed apparent drought tolerance with vigorous growth, whereas the control trees displayed stress symptoms of drying and falling leaves and less root biomass (Figure 2A and 2B). Measurements of plant height revealed a 27.43% increase in transgenic lines under normal conditions [70% field capacity (FC)] and 23.68–50.44% under stress conditions (50% or 30% FC) compared with the control line (Table 1). The transgenic lines also showed significant increases of basal diameter of 84.02% and 57.04% under normal and stress conditions, respectively, relative to control plants (Table 1). The average leaf area of the D5-21 line was significantly greater than that of the control plants under both normal and stress conditions (Table 1). The transgenic lines also showed significant increases of total biomass, ∼28.09% under control conditions and 24.95–67.13% under stress conditions compared with the control line (Table 1). Root, shoot and leaf biomass under non-stressed conditions are listed in Table S2.

**Table 1:** Growth of poplar transgenic lines under drought stress.

		Line		
Growth parameter	FC (%)	Control	D5-20	D5-21
HG (cm)^a^	70	17.50±1.88	23.00±1.69*	21.60±1.14
	50	12.67±0.33	16.00±1.53*	15.67±1.45
	30	9.02±0.21	13.33±0.21*	13.57±0.57*
BDG (mm)^a^	70	0.97±0.03	1.77±0.08*	1.80±0.08*
	50	0.85±0.06	1.16±0.12*	1.25±0.18*
	30	0.56±0.08	1.00±0.19*	0.93±0.05*
LA (cm^2^)^b^	70	91.31±6.19	116.07±2.72	127.06±13.36*
	50	65.82±1.49	83.39±7.75	102.74±4.10*
	30	62.14±6.01	82.63±10.77	97.50±5.09*
TB (g)^c^	70	36.85±2.36	48.61±2.26*	45.77±7.26*
	50	27.53±0.27	35.52±2.80*	34.40±3.69*
	30	18.04±1.08	30.15±2.30*	25.41±1.16*

Height growth (HG, cm), basal diameter growth (BDG, mm), leave area (LA, cm^2^) and total biomass (TB, g) were determined. Means ± SE are shown. Within a treatment (row), means followed by an asterisk are significantly different from the control (*P < 0.05).

a expressed as increase of value after treatment relative to that at beginning of treatment, n  =  10 plants for each line.

b n  =  9 experiments for each line.

c n  =  9 plants for each line.

To investigate the long-term effects of multiple genes on physiological processes, instantaneous water use efficiency (WUEi), maximum quantum yield of photosystem II (PSII) (*Fv*/*Fm*), and levels of fructan, chlorophyll and proline were measured in fresh leaves of transgenic and control plants at the end of the growing season (day 76 of the drought study). While all plants had similar WUEi at 70% FC, the two transgenic lines had significantly higher WUEi than control plants under drought conditions (50% or 30% FC). Both D5-20 and D5-21 transgenic lines showed increased WUEi at 50% or 30% FC (Figure 2C). The D5-20 and D5-21 lines also had higher concentrations of fructan than the control under both normal and stress conditions (Figure 2C). The transgenic poplar plants had higher total chlorophyll in fresh leaves as well as larger leaf areas than the controls under drought conditions (50% or 30% FC) (Figure 2C, Table 1). Unlike the other three measured physiological parameters, proline levels were only significantly higher for transformants when the most severe drought stress (30% FC) was applied (Figure 2C). No significant difference of *Fv*/*Fm* was scored under the three levels of drought treatments.

### Salt tolerance of multigene transformed poplar

**Figure 3:**
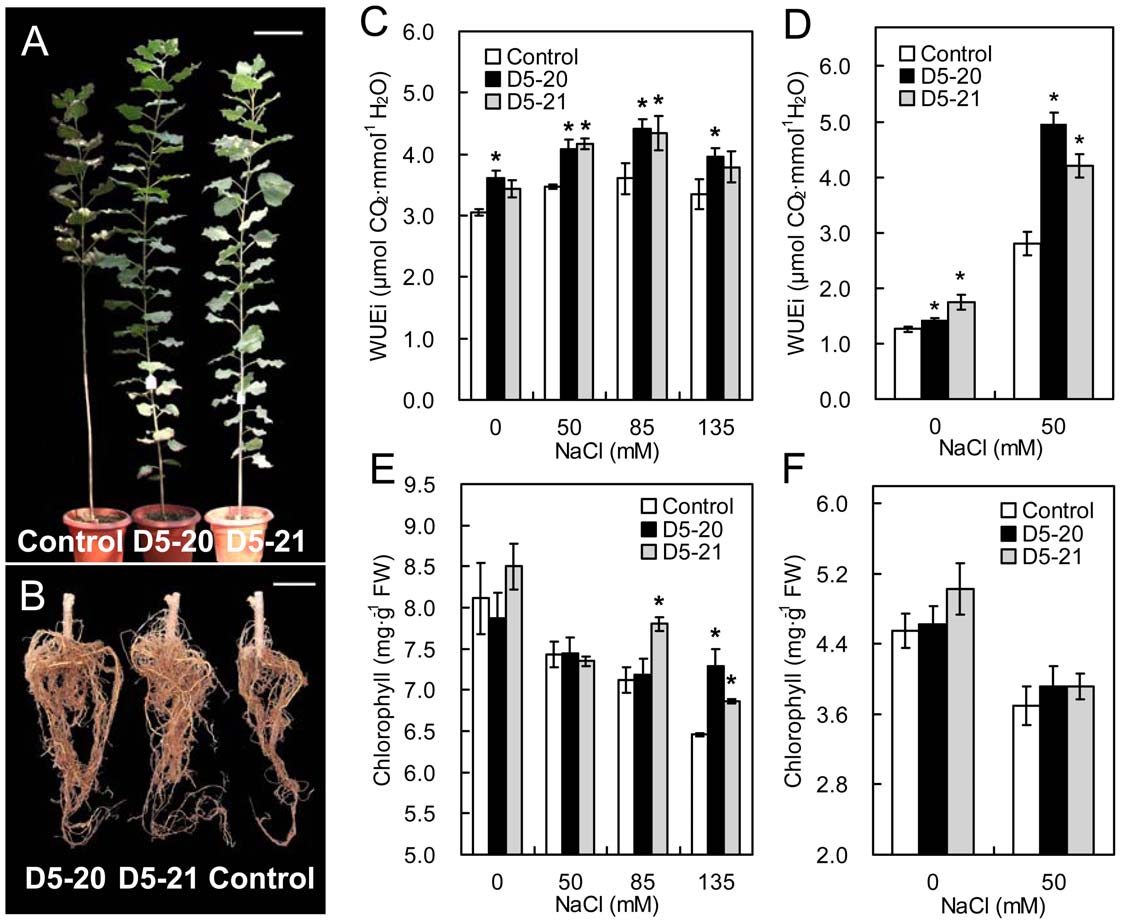
Enhanced salinity resistance of transgenic poplar. Transgenic lines showed more vigorous growth (A) with more developed root systems (B) compared to the control after 78 days of salt stress (135 mM NaCl). Only one representative tree for each line is shown. Bar: 20 cm (A), 10 cm (B). Comparisons of instantaneous water use efficiency (WUEi) in transgenic lines and the control at day 40 (C) and day 78 (D) of NaCl treatments, n  =  9 plants. Comparisons of chlorophyll contents of fresh leaves from the transformants and the control at day 40 (E) and day 78 (F) of NaCl treatments, n  =  9 experiments. FW  =  fresh weight. Data are means ± SE. Asterisks indicate significant differences in the mean compared to the control (* *P* < 0.05).

Physiological parameters for each treatment were measured at the midpoint of the experiment (day 40) when the leaves of some plants were observed to be yellowing and exhibiting signs of chlorosis. It should be noted that only data from the lines exposed to 0 and 50 mM NaCl were obtained at the termination of the experiment (day 78) because of the substantial loss of leaves in the lines tested with 85 or 135 mM NaCl treatments. For this same reason, biomass data was collected without considering the contribution of leaves at the end of the experiment. Under salt stress, transgenic lines showed mild stress symptoms and had more and longer adventitious roots than the control (Figure 3A and 3B). The transgenic trees exhibited an average increase in height of 87.18% under normal conditions (0 mM NaCl) and 46.16–207.10% under salt stress conditions (50, 80, or 135 mM NaCl) (Table 2). The basal diameter also significantly increased to 70.32% greater than that of the control line upon exposure to 135 mM NaCl (Table 2). The improved growth in plant height and basal diameter resulted in a significant increase in stem biomass under salt stress conditions (50, 80, or 135 mM NaCl). Under moderate (80 mM NaCl) and severe (135 mM NaCl) stress conditions, transgenic lines accumulated more root biomass than the control.

**Table 2:** Growth of poplar transgenic lines under salt stress.

		Line		
Growth parameter	NaCl (mM)	Control	D5-20	D5-21
HG (cm)^a^	0	28.00±5.48	51.29±5.37*	53.71±8.23*
	50	19.67±5.07	28.75±3.79*	37.83±3.01*
	80	10.00±2.56	25.25±2.64*	30.71±2.28*
	135	3.14±0.26	4.80±1.83*	5.00±0.45*
BDG (mm)^a^	0	2.27±0.05	2.26±0.09	2.20±0.15
	50	0.81±0.08	1.59±0.19*	1.07±0.09
	80	0.57±0.08	0.98±0.17*	0.61±0.12
	135	0.32±0.06	0.60±0.18*	0.49±0.06*
SB (g)^b^	0	15.39±0.57	22.47±2.05*	20.76±0.57
	50	12.34±0.40	17.56±0.50*	15.92±0.93*
	80	7.54±0.44	11.60±1.11*	10.70±0.49*
	135	6.29±0.69	8.97±0.62*	8.07±0.44*
RB (g)^b^	0	5.14±0.41	6.05±0.28	5.81±0.34
	50	2.31±0.18	3.02±0.35	2.51±0.08
	80	0.92±0.16	2.51±0.29*	1.98±0.03*
	135	0.15±0.01	1.44±0.49*	1.19±0.09*

Height growth (HG, cm), basal diameter growth (BDG, mm), stem biomass (SB, g) and root biomass (RB, g) were determined. Means ± SE are shown. Within a treatment (row), means followed by an asterisk are significantly different from the control (*P < 0.05).

a expressed as increases of value after treatment relative to that at beginning of treatment, n  =  12 plants for each line.

b n  =  9 plants for each line.

On day 40, WUEi values gradually rose with increasing concentrations of salt treatments from 0 to 85 mM NaCl, and decreased at 123 mM NaCl treatment, altogether with generally higher values for the transgenic lines (Figure 3C). On the last test day (day 78) of the 50 mM NaCl treatment, the WUEi levels of the multigene lines were similar to those on day 40 (Figures 3C and 3D). The WUEi of non-transgenic plants, however, declined to about 80.74% of the 40 day level (2.801±0.213 versus 3.469±0.040 µmol CO_2_·mmol^−1^ H_2_O). Chlorophyll concentrations showed an overall downward trend with increasing salt stress, while higher values in the transgenic lines were apparent with 135 mM NaCl treatment (day 40, Figure 3E). When exposed to 50 mM NaCl, the chlorophyll concentration of the multigene-transformed poplar tended to be higher relative to the control plants, but it was not significantly different at test termination (Figure 3F). However, the D5-20 and D5-21 transgenic plants showed no obvious differences from the control plants in proline content and *Fv*/*Fm* under normal and all stress conditions.

### Tolerance to excessively wet (waterlogging) conditions

**Figure 4:**
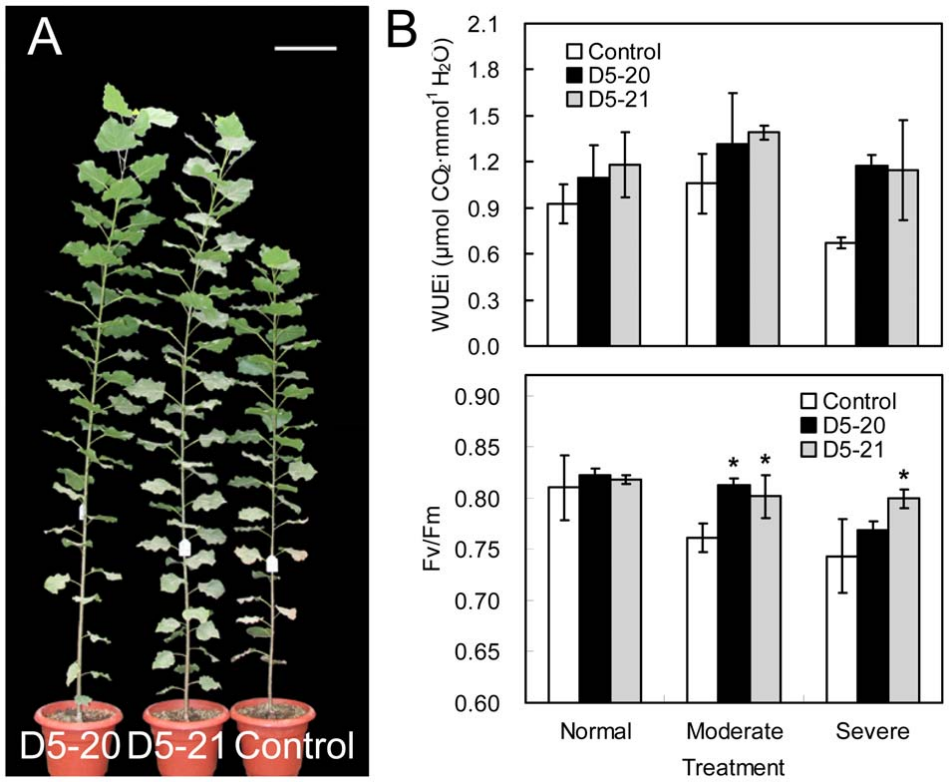
Waterlogging tolerance of transgenic poplar. (A) The transgenic lines displayed less reduction in growth than the control after 98 days of moderate waterlogging stress. Only one representative tree for each line is shown. Bar  =  20 cm. (B) Comparison of instantaneous water use efficiency (WUEi) (upper, n  =  9 plants) and maximum quantum yield of PSII *Fv*/*Fm* (lower, n  =  9 plants) between transgenic lines and the control at day 98 of waterlogging stress. FW  =  fresh weight. Data are means ± SE. Asterisks indicate significantly different values between the transgenic lines and the control (* *P* < 0.05).

When the soil was flooded, the transformants exhibited a waterlogging tolerance phenotype with less influenced vegetative growth than control plants (Figure 4A). No significant difference in root growth of transgenic trees was found compared with the control. Under waterlogging (moderate or severe) conditions, the multigene-transformed D5-21 line showed a 18.57–51.19% higher height, had a 31.52–64.79% greater basal diameter and exhibited 127.06–156.73% higher total biomass in comparison to non-transgenic trees (Table 3). The D5-20 line also displayed better growth parameters than the control under moderate stress conditions (Table 3). Root, shoot and leaf biomass under non-stressed conditions are listed in Table S2.

**Table 3:** Growth of poplar transgenic lines under waterlogging stress.

		Line		
Growth parameter	Treatment	Control	D5-20	D5-21
HG (cm)^a^	Normal	97.83±3.40	95.11±2.77	116.00±0.88*
	Moderate	83.00±4.67	120.94±3.84*	119.56±3.85*
	Severe	42.33±1.76	72.61±5.63*	64.00±3.38*
BDG (mm)^a^	Normal	4.02±0.45	4.11±0.28	5.40±0.14*
	Moderate	3.87±0.48	5.88±0.52*	5.09±0.47*
	Severe	3.55±0.32	4.76±0.62	5.85±0.36*
TB (g)^b^	Normal	19.81±1.65	35.12±3.29*	47.73±2.41*
	Moderate	18.33±1.23	56.87±6.07*	41.62±3.30*
	Severe	16.50±4.41	28.59±3.94	42.36±3.27*

Height growth (HG, cm), basal diameter growth (BDG, mm), and total biomass (TB, g) were determined. Means ± SE are shown. Within a treatment (row), means followed by an asterisk are significantly different from the control (* *P* < 0.05).

a expressed as increases of value after treatment relative to that at beginning of treatment, n  =  9 plants for each line.

b n  =  9 plants for each line.

In contrast to the results from the drought and salt experiments, the transgenic lines did not show significantly higher WUEi than that of the control under waterlogging (moderate or severe) stress (Figure 4B). However, when moderate waterlogging treatment was applied, *Fv/Fm* values were significantly higher in the transgenic lines relative to the control; and the D5-20 line showed greater values for *Fv*/*Fm* than that of the control under the most severe stress conditions (Figure 4B). Both transgenic and control lines had similar chlorophyll contents and proline levels in the waterlogging experiments.

### Insect resistance determined by feeding assays

After seven days of feeding bioassays, the total mortality of *Plagiodera versicolora* larvae fed with transgenic D5-21 leaves was found to be significantly higher than that of the control (56.03±4.51% versus 30.79±4.22%, *P* < 0.05) (Table 4). On day 3 and 5 from the first feeding, the exuviation indices of *P. versicolora* were significantly different between larvae fed with D5-21 and control leaves (0.78±0.12 versus 1.22±0.04 on day 3; 1.73±0.31 versus 3.15±0.26 on day 5, *P* < 0.05) (Table 4).

**Table 4:** Total mortality rates and exuviation indices of *P. versicolora* fed with leaves of transgenic poplar (D5-21) or the non-transgenic control line.

		Exuviations index	
Line	Total mortality rate (%)	Day 3	Day 5
Control	30.79±4.22	1.22±0.04	3.15±0.26
D5-21	56.03±4.51*	0.78±0.12*	1.73±0.31*

Means ± SE are shown. A mean value followed by an asterisk is significantly different from the control (*P < 0.05). Data were arcsin transformed before the analysis of variance.

### Effects of multigenes on insect community and growth performance in field trials

The following insect orders were identified in the field trials in 2006 and 2007: Lepidoptera (Lymantriidae, Notodontidae, Limacodidae), Coleoptera (Chrysomelidae and Coccinellidae), Homoptera (Aleyrodidae and Cicadellidae), Heteroptera (Lygaeidae and Aphidoidea), Neuroptera (Chrysopidae), Hymenoptera (Tenthredinidae and Chalalcididae) and Orthoptera (Acrididae). Representatives of the arachnid order Araneida were also identified. During the 2-year monitoring period, 5,614 arthropod individuals were observed in the field trial, with 3,145 on the control trees and 2,469 on D5-21 transgenics. Community-level analysis showed that the dominance of targeted insects on D5-21 trees was substantially lower than that of control (Table 5). The dominance of non-targeted defoliators (mainly Lepidoptera and Homoptera) on D5-21 was similar to that of the control, whereas the dominance of sucking pests was dramatically increased on D5-21 (Table 5). A lower value of Shannon-Wiener index (*H*') and dominance concentration index (*C*) and higher value of evenness index (*J*) were found on the Arthropod community and pest subcommunity in transgenic trees than the control, while the natural enemy subcommunity displayed a substantially greater *C* value than that of control trees (0.1836 versus 0.1385, Table 6).

**Table 5:** The Berge-Parker dominance index (*I*) of different insect class dominance in the arthropod community of transgenic (D5-21) and non-transgenic poplar.

Line	Targeted insects	Non-targeted defoliator	Sucking pest
Control	0.2254	0.4459	0.0765
D5-21	0.1422	0.4689	0.1143

**Table 6:** Main indices of arthropod community diversity in transgenic (D5-21) and non-transgenic poplar.

		Line	
Type of community	Indices	Control	D5-21
Arthropod community	*H*'	3.2514	3.1363
	*J*	0.7762	0.7856
	*C*	0.0700	0.0657
Pest subcommunity	*H*'	2.7167	2.6804
	*J*	0.7223	0.7369
	*C*	0.1113	0.1007
Natural enemy subcommunity	*H*'	2.3061	2.0839
	*J*	0.7698	0.7209
	*C*	0.1385	0.1836

*H*', Shannon-Wiener index; *J*, evenness index; and *C*, dominance concentration index.

To assess the tolerance of multigene-transformed poplar trees to long-term salt stress under natural conditions, growth of the 2.5-year-old plants from another field trial was measured in September 2007. Although they were raised in high salinity soil, the transgenic trees grew normally and showed no symptoms of salt damage. Compared to the control plants, the transgenic lines had an average 3.82% increase on tree height and 4.12% greater diameter at breast height (DBH) compared with the controls (Table 7), although these increases were not significant.

**Table 7:** Growth of transgenic and control poplar trees in a long-term salinity tolerance field study.

Line	Height (m)		DBH (cm)	
	Mean ± SE	Difference from Control (%)	Mean ± SE	Difference fromControl (%)
Control	8.03±0.54	―	8.63±0.59	―
D5-20	8.52±0.26	6.10	9.17±0.23	6.26
D5-21	8.15±0.27	1.49	8.80±0.37	1.97

Height and diameter at breast height (DBH) were measured in 2.5-year-old trees. Means ± SE are shown.

## Discussion

Previous reports suggested that transfer and expression of four out of the five genes used in this study, alone or in combination, could enhance the ability of trees or other plant species to tolerate certain stress factors. Ectopic expression of *SacB* has been shown to confer drought tolerance in tobacco [25], sugar beet [6] and poplar [26]. *JERF36* was found to be involved in salt resistance in hybrid poplar (*Populus alba* × *Populus berolinensis*) [27]. Transgenic poplar (*P. alba* × *Populus glandulosa*) expressing *vgb* grew better than non-transformed trees under waterlogging stress, as indicated by higher biomass [28]. Génissel *et al*. [29] had reported that total soluble proteins in the leaves of transgenic *Populus tremula* × *P. tremuloides* transformed with a synthetic *Cry3A*(*a*) gene caused significant lethality to the poplar leaf beetle, *Chrysomela tremulae*. Resistance to insects was also conferred by the expression of *BtCry3A* and *OC-I* [30]. In the current study we demonstrated that the combined expression of these five genes (*SacB*, *JERF36*, *vgb*, *BtCry3A* and *OC-I*) conferred tolerance to drought, salt, and waterlogging, as well as resistance to insects in *P*. × *euramericana* ‘Guariento’. Our approach resulted in a convergence of several genes involved in multiple pathways to improve stress tolerance phenotypes, which could not be achieved by single gene transformation studies. The combined effects from multiple genes resulted in highly complex traits, and future studies comparing these multigene transformed poplars with individual gene transformants may help to better identify the relationships between the traits and the transgene functions.

At the end of the drought and salt stress period, the transgenic lines were found to have better growth than the control, as shown by greater height, basal diameter and biomass. This improved growth could be primarily attributed to higher WUEi for the D5-20 and D5-21 transgenic lines (Figures 2C and 3C), since it was an important indicator of plant yield [31], [32]. Fructan levels in the transformants were significant higher than the controls under drought conditions. These results were consistent with other works showing that sugar beet or tobacco transformed with *SacB* also displayed increased levels of fructan [3], [25]. It was proposed that fructan could protect membranes by interacting with lipids and phospholipids [33], [34]. Although previous reports demonstrated that *SacB* was involved in drought tolerance, the enhanced salt resistance phenotype has not yet been observed in *SacB*-transformed plants [25]. A previous study showed that expression of *SacB* also increased the levels of total non-structural carbohydrates (glucose, fructose, sucrose, starch, and fructan) in potato [35]. Since other non-structural carbohydrates could be related to osmotic stress tolerance [3], the potential increase in total non-structural carbohydrates in the transgenic poplar trees should to be evaluated in the future. On the other hand, our previous work revealed that the introduction of *JERF36* into poplar enhanced salt tolerance in both greenhouse and field experiments [27], and we proposed that expression of *JERF36* was the major contributor to the phenotype. In this study, the D5-20 and D5-21 transgenic lines displayed increased WUEi and root biomass, which suggested that *JERF36* could regulate plant WUE and root growth under salt stress. Although higher WUEi and better root growth were also observed on transgenic plants under drought stress, whether *SacB* was involved in plant WUE regulation or root architecture remained to be determined since potential effects on drought tolerance of *JERF36* could not be unambiguously interpreted from the present data. Although no previous data are available on the function of *JERF36* under drought conditions, recent work by Wu *et al*. [36] suggested that another *ERF* gene, *JERF3*, could confer drought tolerance in transgenic tobacco. While this finding implied that *JERF36* may have play a significant role in drought tolerance in this study, the assumed interaction or crosstalk between *JERF36* and *SacB* in multigene transformed poplar requires further investigation.

Proline accumulation under abiotic stress conditions has been correlated with protection of subcellular structures by osmotic adjustment [37], [38] and free radical detoxification [39]. Increased proline was also shown to play a role in enhancing photosynthesis efficiency [40]. In this study, higher concentrations of free proline were apparent in the transformants at the end of the drought experiments (Figure 2C), which implied that proline biosynthesis in transgenic plants may have been regulated by at least one transgene. Moreover, similar levels of total chlorophyll were detected in both transgenic and control plants in the waterlogging test treatment even after 98 days of exposure (data not shown), which suggested that the combined expression of the five genes had little effect on long-term chlorophyll synthesis during extended waterlogging stress.

The observed improvement in waterlogging tolerance of poplar could be partly explained by the elevated *Fv*/*Fm* value, which was not observed in either the drought or salt stress experiments. *Fv*/*Fm* values have been shown to be stable in any given plant species under non-stressed conditions [41], and the changes in *Fv*/*Fm* observed in this study may have been a result of altered photosynthetic function. Previous report described that expression of the *Vitreoscilla* hemoglobin gene *vhb* lead to enhanced accumulation of starch in aspen chloroplasts [13]. Therefore, the increased *Fv/Fm* suggested that the presence of *vgb* and its resulting product hemoglobin provide a substantial level of protection for the photosynthetic machinery of transgenic lines against waterlogging stress. The enhanced waterlogging tolerance could also be attributed to the oxygen gathering and delivery functions of hemoglobin which, in turn, benefits cell growth and protein synthesis under oxygen-limited conditions [42], [43]. In our study, under salt stress multigene overexpression resulted in a higher chlorophyll content, which was also observed in the drought experiments. Higher chlorophyll concentrations may have been related to the slower rate of chlorophyll pigment degradation and/or an increased number of photosynthetic mesophyll cells, which has been shown to influence transpiration efficiency [44]. Since stomatal behavior and transpiration efficiency were deemed highly relevant to plant WUE [45], the relationship between these two factors and WUE in multigene tranformants will be explored in future research.

At the end of the greenhouse experiments, trees from at least one transgenic line were unexpectedly larger in size compared with control trees under non-stressed conditions (Tables 1, 2, 3). In the drought stress experiments, both transgenic lines had higher total biomass with 70% FC treatment (Table 1). D5-20 displayed increased shoot biomass under non-stressed conditions in the salt experiments (Table 2), and D5-21 showed increased shoot and leaf biomass under non-stressed conditions in the waterlogging experiments (Table S2). This observation raised the possibility that the expression of transgenes conferred improved growth of shoot or leaf, which may have resulted in indirect effects on the stress tolerance in the transgenic poplar. Thus, additional work is needed to address the mechanisms underlying these effects.

The insect-feeding assays showed higher total mortality rates and lower exuviation indices of leaf beetle (*P. versicolora*) larvae fed with leaves from transgenic trees than those fed with leaves from control trees (Table 4), which was a direct reflection of enhanced resistance of transgenic poplar to the target insects tested. This result was consistent with our population-scale analysis of field trial, which revealed lower dominance of targeted insects (Coleopterar) on the transgenics than on the control trees. This result could be explained by the reduced number of targeted insects in the transgenic trees. The observed effects on pests were also reflected by a slightly decreased *H*' and *C* and increased *J* of pest subcommunity in the D5-21 transgenic line (Table 6). The minor differences of *H*', *C* and *J* for arthropod between the transgenic line and the control also indicated that transformation of multiple resistance genes in poplar did not have a significant negative effect on the arthropod community. However the sucking pests in the D5-21 transgenic line increased, and the reason for this phenomenon remained to be uncovered. In the field trial for salt tolerance, the average tree height and DBH of transgenic lines did increase by 3.82% and 4.12%, respectively, compared with the control. However, these increases were not significant, which could be partly attributed to the potentially non-uniform distribution of soil salinity (especially in the deep soil zone). The resulting variations in stress effects on trees planted in a small area would have weakened the statistical comparisons of effects between transgenic trees and the control. Thus, a field trial with a larger area and longer investigation time (e.g. a rotation for poplar) may be necessary to confirm improved salt tolerance in the multigene transformed trees.

We initially used biolistic bombardment to obtain multiplegene transgenic poplar because most other existing approaches, such as multiple transformations of separate genes or one vector carrying multiple genes using *Agrobacterium tumefaciems* [46], [47], and inter- or intra-specific crosses [48] required substantial commitments of time and effort, particularly when working with tree species. Transformation using biolistic bombardment may be a reasonable approach for trees due to its simplicity and speed. For the purpose of practical and commercially-applicable breeding, precise effects of genes that have been associated with certain stress-tolerance responses need to be assessed over extended periods of time [49], particularly for trees which are larger, and grow much more slowly than typical crop plants. The characteristics manifested by the transgene expression could be obvious early in a plant's life cycle, but it could also only be apparent after months or years of growth. For that reason in the current study, long-term greenhouse and field experiments were used which showed improved tolerance to multiple stressors to a certain extent in the transgenic lines. These results suggest that it may be possible to develop commercially-viable, superior cultivars exhibiting higher tolerance to multiple stressors through the coordinated manipulation of multiple genes. Because of the complex growth and physiologic phenotypes and variability in stress tolerance among transgenic lines, careful research and assessment are required to ensure the long-term sustainability of desirable plant responses.

## Materials and Methods

### Ethics statement

All approvals for field trials of transgenic poplar were obtained from the State Forestry Administration, People's Republic of China, under application number 2005-06.

### Plant material and transgenes

The inter-specific hybrid from *P. nigra* and *P. deltoides*, *P*. × *euramericana* ‘Guariento’ was used in this study. The transgenic poplar lines were produced as described previously [24]. Briefly, four constructs containing *SacB*, *JERF36*, *vgb* and chimeric genes *BtCry3A*+*OC-I* were combined and delivered into the poplar using particle bombardment. All these genes were under the control of the CaMV 35S promoter. Transgenic plants harboring foreign genes were identified by PCR and Southern blotting. Based on our previous study [24], two transgenic lines, D5-20 and D5-21 carrying the five genes mentioned above, and one non-transgenic line (control) were chosen here for further analyses.

### qRT-PCR

Cuttings of the transgenic lines D5-20, D5-21 and the control line were cultured in a greenhouse. When the plants grew to about 20 cm with seven to nine leaves, fully expanded leaves from each line were collected, frozen immediately in liquid nitrogen and stored until use. Total RNA was extracted from leaves using Ambion® Plant RNA Isolation Aid (Applied Biosystems, CA, USA) according to the manufacturer's instructions. cDNA was synthesized using the PrimerScript RT reagent Kit (TaKaRa, Dalian, China). qRT-PCR was performed on an ABI 7500 FAST sequence detector (Applied Biosystems) with SYBR Green Real-time PCR Master Mix (TaKaRa). Gene-specific primers were designed to amplify 120–130 bp fragments of foreign genes, and parallel PCR were carried out using a gene-specific primer pair for poplar *ACTIN1* (GenBank Accession XM_002298674) used as a reference gene. Primer sequences for the real-time PCR assay of the five genes and *ACTIN1* are listed in Table S1. Five trees for each line were tested, and four PCR replicates were performed for each RNA sample.

### Greenhouse stress experiments

Drought resistance, salt tolerance and waterlogging tolerance were assessed in separate greenhouse studies. In March 2007, cuttings of transgenic and control plants were placed in plastic pots (with drain hole in the bottom, 30 cm deep × 25 cm diameter) containing an experiment-specific soil mixture. Drought, salt and waterlogging experiments were conducted, respectively, from on July 30–October 13 (76 days), July 5–September 20 (78 days) and June 4–September 9 (98 days) in 2007. For the drought experiments, pots were filled with a mixture of nursery soil, fine sand and peat (10∶2∶1, v/v). Plants of each line were given one of three drought treatments: 70% FC with gravimetric soil moisture (GSM) maintained at 14.14% which was considered the normal (non-stressed) condition; 50% FC with GSM maintained at 10.10%; and 30% FC with GSM maintained at 6.06%. Each treatment contained ten replicates. The percentages of FC were maintained by weighing pots daily to determine the amount of water to be added. For the salt tolerance experiments, the soil mixture was comprised of peat, perlite and sandy soil (8∶1∶3, v/v). Plants of each line were treated with different concentrations of salt (0, 50, 85 and 135 mM NaCl solution) with twelve replicates per treatment. For the waterlogging experiment, a mixture of peat, perlite and sandy soil (3∶1∶1, v/v) was added to each pot. Plants of each line were exposed to three water:soil treatments: normal (non-stressed) condition (70% FC), moderate stress (saturated soil with the water level at the soil surface, 65.38% GSM) and severe stress (water level at 2 cm above the soil surface). There were nine replicates per treatment. Each pot was placed in a plastic basin to prevent water or salt loss. The amount of water to be added was determined by weighing pots daily, and water was added to each pot twice a day to maintain the designated FC percentage. Tap water was used at the initiation and throughout the experiments.

### Growth and physiological analyses

Growth was determined by measuring the height and basal diameter of each plant at test initiation and termination. To measure biomass, at least three plants from each transgenic or control line from each treatment were sampled. Root, stem and leaves were oven-dried at 105°C for 0.5 h. The plant tissue was left undisturbed for 72 h at 80°C, after which each sample was weighed separately. Leaf area was measured for four leaves of each of three replicate plants in each treatment by weighing a paper copy of each leaf and comparing the weight against a standard weight-area curve.

Gas exchange parameters including net photosynthesis rate (*A*) and transpiration rate (*T*) were measured using the Li-6400 Portable Photosynthesis System (LI-COR, NE, USA) [50]. WUEi for each line was calculated as WUEi = *A*/*T*, which was the amount of carbon gained in photosynthesis in exchange for water used in transpiration [44]. Data were collected on the 6^th^, 7^th^ and 8^th^ leaf from the top of each tree, with three replicates for each treatment. To standardize the data as much as possible, measurements were always made between 08:30 and 11:30 on a cloudless day to avoid the low humidity and high temperature conditions that often occur in the afternoon. Conditions during these measurements were 400 µmol·mol^−1^ of CO_2_, artificial lighting and 800 µmol·m^−1^·s^−1^ photosynthetically active radiation (PAR). The maximum quantum yield (or maximal fluorescence efficiency) of PSII (*Fv*/*Fm*) was measured with a PAM-2100 portable chlorophyll fluorometer (Walz, Effeltrich, Germany) between 08:30 and 11:30 on a sunny day before termination of the stress tests in mid-September [51]. *Fv*/*Fm* was also determined on the 6^th^, 7^th^ and 8^th^ functional leaf for each treatment after at least 20 min of dark adaptation with light-tight leaf clips. Each measurement was repeated three times with three technical replicates.

Fructan levels of leaf tissues were determined for drought experiments using a plant fructan colorimetric assay kit (Cat. No. GMS19024.1, Genmed Scientifics DE, USA) according to the manufacturer's protocol. Free proline was extracted from fresh leaves using 3% 5-sulfosalicylic acid and quantified by the colorimetric method [52]. The chlorophyll concentration was determined from 0.2 g of fresh leaf tissue and extracted in 80% acetone. Absorbance was measured at 647 and 664.5 nm. The concentrations of chlorophyll *a*, chlorophyll *b* and total chlorophyll were calculated by using the following equations: 1) Chl *a*  =  12.63A_664.5_−2.52A_647_, 2) Chl *b*  =  20.47A_657_−4.73A_664.5_ and 3) Total Chl  =  17.95A_647_+7.90A_664.5_; where A is the absorbance in a 1 cm cuvette [53].

### Feeding assay for insect resistance

*P. versicolora* (Laicharting) was chosen as the target pest for feeding experiments, since this insect has shown to be an important source of damage to willow and poplar leaves [54]. The *P. versicolora* egg masses were collected from poplar trees near the Chinese Academy of Forestry and hatched under laboratory conditions at 28–32°C. Healthy one-day-old larvae were fed daily with equal numbers of fresh, clean, fully expanded leaves of D5-21 and non-transgenic control plants. Each treatment had 20 larvae, and the experiment was repeated three times. Dead larvae were removed and recorded daily for a period of seven days. Larval exuviation was monitored three and five days after the first feeding. Total mortality rate and exuviation index were used to evaluate the effects of transgenic poplar on *P. versicolora*:

Total mortality rate  =  (number of dead larvae at the end of feeding/total number of larvae) × 100%;

Exuviation index  =  (first instar × larvae number + second instar × larvae number + … last instar × larvae number)/total number of larvae × 100%.

### Field trials

To test for insect resistance, cuttings of the multigene line D5-21 and a control line studied in the greenhouse experiments were planted in a field at Fangshan, Beijing, on April 2006. One hundred trees of each line were planted in a square (10 rows by 10 columns) with 2.0 m intervals between trees. The taxonomic classifications and number of arthropods on the plants were monitored. During the growing season trees were monitored monthly from June to September in 2006 and from May to September in 2007. Insects were monitored in 20 trees for each line, and a ground survey was also conducted. Four branches from the mid-region and four from the lower region of the tree canopy were evaluated (one from each cardinal compass point), for a total of eight sampled branches.

Pest community was classified in three guilds including targeted insects, non-targeted defoliators and sucking pests, and the Berge-Parker index (*I*, 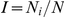) was used to evaluate the level of insect resistance of transgenic trees. The Shannon-Wiener index (*H*', 
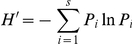
), evenness index (*J*, 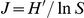) and dominance concentration index (*C*, 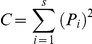) [55] were used to reflect both potential negative effects and resistance of transgenic trees on the arthropod community, pest subcommunity and natural enemy subcommunity. In these indices, *Pi* is the proportion of the *i*th species in the total sample, *S* is the species richness, *N_i_* is the number of *i*th species, and *N* is the total number of all samples in the community.

To evaluate salt tolerance under field conditions, a second trial was established at Shouguang Experiment Station, Shandong province, on March 2006. Established trees (1-year-old) from D5-20 and D5-21 transgenic lines plus one control line were planted in a randomized block design. The field trial consisted of six blocks, each containing three replicates for each line. Rows and trees within rows were 3 m apart. The soil in which the trees were grown was saline alkali. The salt content was 0.2–0.6%, with NaCl accounting for about 80–90% of the total salt load. At the end of the test, the height of the 2.5-year-old trees and diameter at breast height (DBH, 1.3 m above the ground level) were measured.

### Statistical analysis

Differences among the transgenic and control lines for growth, physiological and insect-resistance properties were evaluated by one-way analysis of variance (ANOVA, α  =  0.05) with Duncan's multiple range test used for multiple comparison of means. Data from each ANOVA was checked to confirm that the corresponding assumption was satisfied. Statistical analyses were performed using Data Processing System (DPS) 3.01 software [56].

## Supporting Information

Table S1 Description of primers used in qRT-PCR.
(DOC)

Table S2 Root, shoot and leaf biomass under non-stressed conditions for drought and water logging experiments.
(DOC)
