# Supplementary material for: Expression of Multiple Resistance Genes Enhances Tolerance to Environmental Stressors in Transgenic Poplar (Populus × euramericana ‘Guariento’)
Source: PLoS One. 2011 Sep 9;6(9):e24614. doi: 10.1371/journal.pone.0024614 (PMC3170361; doi:10.1371/journal.pone.0024614)
Supplement: Table S1 — Description of primers used in qRT-PCR. (DOC) [file pone.0024614.s001.doc]

**Table S1** Description of primers used in qRT-PCR.

| Gene | Primer name | Primer sequence (5’→ 3’) |
| --- | --- | --- |
| *vgb* | VgbF | GAAGGAGCATGGCGTTACCAT |
|  | VgbR | TCGCCAAAGCCTTAGGCTG |
| *SacB* | SacBF | AGATGGCTACCAAGGCGAAGA |
|  | SacBR | TTGCTAACTCAGCCGTGCG |
| *BtCry3A* | BtCry3AF | CAGAGGAACCATCCCAGTGCT |
|  | BtCry3AR | AGGCACCAGATTGGAGCTTGT |
| *OC-I* | OC-IF | CGTCACCGAGCACAACAAGA |
|  | OC-IR | GCATCCCCTTCCTTCACCTC |
| *JERF36* | JERF36F | CTCTCAACCCCAAACGAGCTC |
|  | JERF36R | CACGAATTTCAGCAGCCCA |
| *ACTIN1*^a^ | ACTIN1F | CATCCAGGCTGTCCTTTCCC |
|  | ACTIN1R | AACGAAGGATGGCGTGTGG |

^a^ reference gene
