# Supplementary material for: Expression of Multiple Resistance Genes Enhances Tolerance to Environmental Stressors in Transgenic Poplar (Populus × euramericana ‘Guariento’)
Source: PLoS One. 2011 Sep 9;6(9):e24614. doi: 10.1371/journal.pone.0024614 (PMC3170361; doi:10.1371/journal.pone.0024614)
Supplement: Table S2 — Root, shoot and leaf biomass under non-stressed conditions for drought and water logging experiments. (DOC) [file pone.0024614.s002.doc]

Table S2 Root, stem and leaf biomass under non-stressed conditions for drought and water logging experiments.

|  | Line | | |
| --- | --- | --- | --- |
| Parameter | Control | D5-20 | D5-21 |
| RB (g)^a^ | 4.57±0.33 | 4.62±0.55 | 4.67±0.26 |
| SB (g)^a^ | 18.40±1.53 | 27.43±2.56 | 25.21±1.64 |
| LB (g)^a^ | 13.88±0.53 | 16.55±0.59 | 15.90±0.40 |
| RB (g)^b^ | 1.84±0.34 | 2.85±0.34 | 4.53±0.84 |
| SB (g)^b^ | 10.90±1.19 | 19.78±2.21 | 26.83±1.13* |
| LB (g)^b^ | 7.07±0.24 | 12.49±1.03 | 16.37±0.95* |

Root biomass (RB), stem biomass (SB) and leaf biomass (LB) were recorded under non-stressed conditions. Means ± SE are shown. Within a row, a mean value followed by an asterisk is significantly different from the control (* *P* < 0.05).

^a^ data were obtained from plants growing in non-stressed conditions (70% FC) in the drought experiment.

^b^ data were obtained from plants growing in non-stressed conditions (70% FC) in the waterlogging experiment.
